# Supplementary material for: The Shear Stress–Regulated Expression of Glypican-4 in Endothelial Dysfunction In Vitro and Its Clinical Significance in Atherosclerosis
Source: Int J Mol Sci. 2023 Jul 18;24(14):11595. doi: 10.3390/ijms241411595 (PMC10380765; doi:10.3390/ijms241411595)
Supplement: Supplementary file 1 [file ijms-24-11595-s001.zip › ijms-2462466-supplementary.pdf]

Table S1. Antibodies used for flow cytometry

Antibodies	Isotype	Conjugate	Supplier<br>Catalog No.
**Isotype control**			
Mouse IgG_1 κ	-	FITC	BD Bioscience<br>555909
Mouse IgG_1 κ	-	PE	BD Pharmingen ™<br>555749
Mouse IgM κ	-	None	Biolegend<br>401601
Rabbit polyclonal	-	None	Biolegend<br>910801
**Primary antibodies**			
Anti-CD31/PCAM-1	Mouse IgG_1	FITC	BD Pharmingen™<br>555445
Anti- CD54/ICAM-1	Mouse IgG_1	PE	BD Bioscience<br>555511
Anti-CD62P/P-selectin	Mouse IgG_1	FITC	BD Pharmingen™<br>555523
Anti-CD62E/E-selectin	Mouse IgG_1	PE	BD Pharmingen™<br>551145
Anti-CD45	Mouse IgG_1	FITC	BD Pharmingen™<br>555482
Anti-CD105/Endoglin	Mouse IgG_1	PE	R&D Systems<br>FAB10971P
Anti-CD106/VCAM-1	Mouse IgG_1	PE	BD Pharmingen™<br>555647
Anti-heparan sulfate (10E4)	Mouse IgM κ	None	Amsbio<br>370255-1
Anti-glypican-4	Rabbit<br>polyclonal	None	Acris<br>AP02029SU
Anti-chondroitin sulfate (CS-56)	Mouse IgM	None	ThermoScientific<br>MA1-83055
Anti-biglycan	Rabbit<br>polyclonal	None	ThermoScientific<br>PA5-13700
**Secondary antibodies**			
Anti-mouse IgM	-	FITC	BioLegend®<br>406506
Anti-rabbit IgG	-	PE	BioLegend®<br>406421

Table S2. Correlation matrix between MMP2 and HSPG/CSPG genes in fold differences on day 7 over day 0

Group	BGN	GPC1	GPC3	GPC4	HSPG2	MXRA5	SDC1	SDC2	SDC4
**HUVECs without TNF-α stimulation**	0.64	-0.15	0.11	0.64	0.45	0.10	0.05	0.88	0.40
**p-value**	0.062	0.685	0.802	0.097	0.230	0.832	0.952	0.003*	0.297
**HUVECs with TNF-α stimulation**	0.55	0.15	-0.02	0.07	0.34	-0.24	0.30	0.46	0.42
**p-value**	0.128	0.699	0.955	0.863	0.372	0.542	0.428	0.211	0.265
**HUAECs without TNF-α stimulation**	0.81	-0.56	-0.04	0.30	-0.22	0.59	-0.39	0.65	-0.04
**p-value**	0.009*	0.120	0.923	0.429	0.563	0.095	0.306	0.059	0.912
**HUAECs with TNF-α stimulation**	0.95	0.10	0.13	-0.35	0.61	-0.42	0.52	0.40	0.40
**p-value**	0.000*	0.997	0.834	0.393	0.148	0.850	0.310	0.276	0.311

BGN, biglycan; GPC1, glypican-1; GPC3, glypican-3; GPC4, glypican-4; HSPG2, heparan sulphate proteoglycan-2; HUAECs, human umbilical artery endothelial cells; HUVECs, human umbilical vein endothelial cells; MXRA5, matrix-remodeling-associated protein 5; SDC1, syndecan-1; SDC2, syndecan-2; SDC4, syndecan-4; TNF-α, tumor necrosis factor-α.
* significant difference

Table S3. Correlation matrix between GPC4 and genes for cholesterol transport in fold changes from day 0 to day 7 and differenced after TNF-α stimulation

Group	ABCA1	ABCG1	LCAT	LDL-R	PCSK9-	VLDL-R
Fold changes from day 0 to day 7						
HUVECs without TNF-α	0.02	0.12	0.55	0.25	-0.23	0.08
p-value	0.612	0.644	0.052	0.408	0.585	0.753
HUVECs with TNF-α	-0.64	-0.52	0.44	-0.57	-0.36	0.05
p-value	0.063	0.155	0.237	0.107	0.348	0.893
HUAECs without TNF-α	0.36	0.68	0.22	-0.23	-0.04	0.33
p-value	0.348	0.042*	0.565	0.558	0.927	0.382
HUAECs with TNF-α	0.22	-0.11	0.05	-0.50	-0.52	0.45
p-value	0.596	0.795	0.914	0.211	0.189	0.263
Fold changes after TNF-α stimulation						
HUVECs day 0	0.36	0.11	-0.42	0.29	-0.60	-0.01
p-value	0.342	0.771	0.264	0.444	0.086	0.975
HUVECs day 7	-0.07	0.12	0.39	0.03	-0.14	0.60
p-value	0.838	0.759	0.297	0.970	0.742	0.097
HUAECs day 0	-0.60	-0.70	0.62	-0.67	-0.55	-0.70
p-value	0.089	0.034*	0.076	0.047*	0.125	0.035*
HUAECs day 7	0.18	0.58	0.39	-0.03	0.47	0.22
p-value	0.672	0.129	0.342	0.949	0.241	0.599

ABCA1, ATP binding cassette subfamily A member 1; ABCG1, ATP binding cassette subfamily G member 1; HUAECs, human umbilical artery endothelial cells; HUVECs, human umbilical vein endothelial cells; LCAT, lecithin-cholesterol acyltransferase; LDL-R, low-density lipoprotein receptor; PCSK9, proprotein convertase subtilisin kexin type 9; VLDL-R, very low-density lipoprotein receptor. * significant difference

Table S4. Primers used for qPCR analysis

Function	Gene	mRNA sequence accession number	Primer sequence	Amplicon
Sulfotransferase	CHST7	NM_019886.3	F: ATGCGTCTGCTCGCCTAC<br>R: GAGAGTGTGACAGATTGCCCC	216
	CHST15	NM_015892.4	F: CTGGACCTCTTTGACCTGGC<br>R: CCATCCGTGCTGTTGTCGTA	164
HSPC or CSPG	GPC1	NM_002081.2	F: AGAAGCTGGTCTCCGAAGC<br>R: ACCTCGGGGTTGTTGATCTG	199
	GPC3	NM_001164617.1	F: CCAAAAGGCAGCAAGGAATGG<br>R: ACCGCAGTCTCCACTTTCAA	199
	GPC4	NM_001448.2	F: GTCTCGTGACTGTGAAGCCA<br>R: TCTGCCACCATCAGCATAGC	124
	HSPG2	NM_001291860.1	F: CAGCATCTCAGGAGACGACC<br>R: CTCCAGCGTGTCTACCACAG	175
	BGN	NM_001711.5	F: GACAACAACAAGTTGGCCAGG<br>R: GACAGAAGTCGTTGACACCCA	112
	MXRA5	NM_015419	F: ATCCTTGTGCCTGCTACGTC<br>R: TCCAACTTGGTCAGTCCTGC	160
	SDC1	NM_001006946.1	F: GGTGCTGGGAGGGGTCATTG<br>R: TTGTTTCGGCTCCTCCAAGG	127

	SDC2	NM_002998.3	F: GACTACGCTTCTGCGTCTGG<br>R: TATTCAGCGTCGTGGTTTCCA	130
	SDC4	NM_002999.3	F: ACAAGGTGTCAATGTCCAGCA<br>R: ACGATGCCACCCACAATCAG	88
Degradation enzyme	HSPE1	NM_006665.5	F: TGGCACCAAGACAGACTTCC<br>R: AGGGCCATTCCAACCGTAAC	152
	GALNS	NM_000512.4	F: AGCTCAACGTGTGCAACTGG<br>R: AGAGGCACTTCTTGGGAATGG	105
	MMP2	NM_001127891	F: GTGGATGCCGCCTTTAACTG<br>R: TTCCAGGCATCTGCGATGAG	131
Collagen	COL4A5	NM_000495.4	F: TTCAAGCATTGGTCTTCCAGG<br>R: CCAGGAGGGCCACTAATACC	210
Cholesterol transport	LCAT	NM_000229.1	F: AAGGACCGCTTTATTGATGGC<br>R: TGGTGGTTATGCGCTGCTC	148
	ABCA1	NM_005502.3	F: TCTGAGCTTTGTGGCCTACC<br>R: CTTGCTCGGGAAGGGAGATG	123
	ABCG1	NM_004915.3	F: AGCGCCAAACTCTTCGAGC<br>R: CAGCCGACTGTTCTGATCACC	192
	LDL-R	NM_000527.4	F: ACTCGCTGGTGACTGAAAACA<br>R: GGCGGTTGGCACTGAAAATG	243
	VLDL-R	NM_003383.4	F: GTGCACAAATGGTCGCTGTA<br>R: TTCCATCGGCTGGGAACAC	153
	PCSK9-2	NR_110451.1	F: TCAAGGAGCATGGAATCCCG<br>R: ACACACGTGTTGTCTACGGC	136

Non-sulfated GAG	LYVE1	NM_006691.3	F: TCCAGTGAGCCGACAGTTTG<br>R: GCCACCGAGTAGGTACTGTC	162
	CD44	NM_000610	F: GGACAAGTTTTGGTGGCACG<br>R: TCCGTCCGAGAGATGCTGTA	141
	HYAL1	NM_153285.2	F: TGGGAAAATACAAGAACCAAGGAAT<br>R: TCACGTTCAGGATGAAGGGC	85
	HYAL2	NM_003773.4	F: CGGGGCTTAGTGAGATGGAC<br>R: GTCTCCGTGCTTGTGGTGTA	109
Housekeeping	YWHAZ	NM_003406.3	F: AACAGCAGATGGCTCGAGAA<br>R: GCAACCTCAGCCAAGTAACG	175

Table S5. Antibodies used for immunofluorescent staining

Antibodies	Isotype	Dilution	Supplier Catalog No.
**Primary antibodies**			
Anti-glypican-4	Rabbit polyclonal	1:100	Acris/Origene AP02029SU-N
Anti-heparan sulfate	Mouse polyclonal	1:100	Amsbio 370255-S
Anti-Δ-heparan sulfate	Mouse monoclonal	1:100	Amsbio 370260-1
Anti-VCAM-1 (BBIG-V1)	Mouse monoclonal	1:100	R&D Systems BBA5
Anti-E-selectin (BBIG-E4)	Mouse monoclonal	1:100	R&D Systems BBA13
**Secondary antibodies**			
Anti-rabbit IgG-Alexa 488	-	1:500	Molecular Probes A11008
Anti-mouse IgG-Alexa 555	-	1:500	Molecular Probes A21422

Table S6. Antibodies used for immunohistochemistry staining

Antibodies	Isotype	Dilution	Supplier<br>Catalog No.
**Primary antibodies**			
Anti-Glypican-4	Rabbit polyclonal	1:800	Acris/Origene<br>AP02029SU-N
Anti-heparan sulfate	Mouse polyclonal	1:200	Amsbio<br>370255-S
Anti-syndecan-4	Rabbit polyclonal	1:800	Biozol
Anti-CD68	Mouse polyclonal	Ready to use	Agilent
**Secondary antibodies**			
Goat-anti-rabbit-Ig	polyclonal	1:800	Agilent
Rabbit-anti-mouse-Ig	-	Ready to use	Agilent

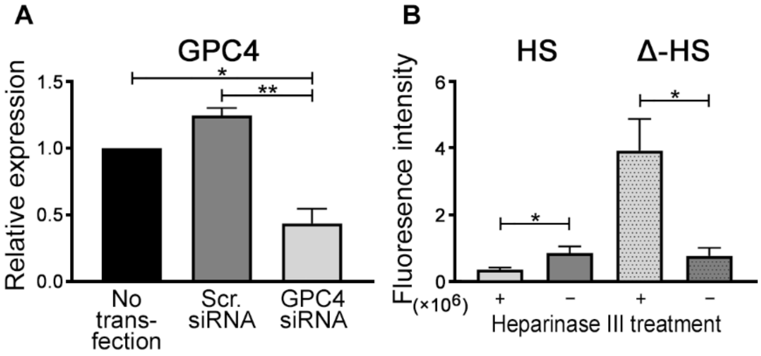

Figure S1. Verification of GPC4 and HS disruption

Relative mRNA expression of GPC4 in siRNA-transfected HUVECs over non-transfected HUVECs (*n* = 3) (A). GPC4 mRNA was significantly reduced in HUVECs transfected with GPC4 siRNA compared with those without transfection ($p < 0.05$) or those transfected with scrambled siRNA (1-way ANOVA with repeated measures with Holm-Sidak post hoc test; $p < 0.01$).

Verification of heparinase III treatment in HUVECs under laminar flow conditions (*n* = 4) (B). By heparinase III treatment, fluorescence intensities for HS signaling was reduced (left, paired t-test; $p < 0.05$) and those for ΔHS signaling was increased (right, paired t-test; $p < 0.05$), verifying the HS degradation by heparinase III. Data are expressed as mean ± SEM. * $p < 0.05$, ** $p < 0.01$. GPC4: glypican-4, HS: heparan sulfate, Scr. siRNA: scrambled small interfering RNA.

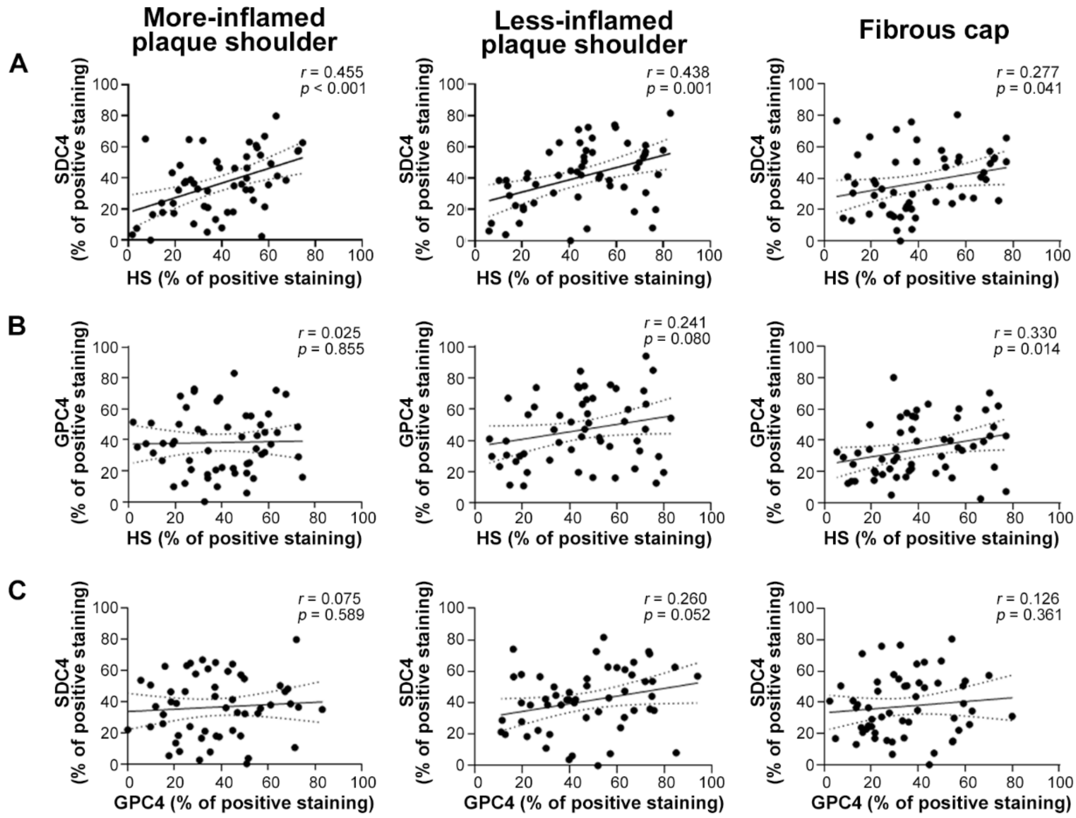

Figure S2. Correlation analysis between the GCX-components in the two PSs and the FC in all plaque sections

Pearson correlation was done between SDC4 and HS (A), GPC4 and HS (B) and SDC4 with GPC4 (C) of the percentage results described in Figure 7. Graphs showing simple linear regression with 95% confidence interval (dotted line) r: Pearson coefficient, PS: plaque shoulder, GPC4: glypican-4, HS: heparan sulfate, SDC4: syndecan-4.

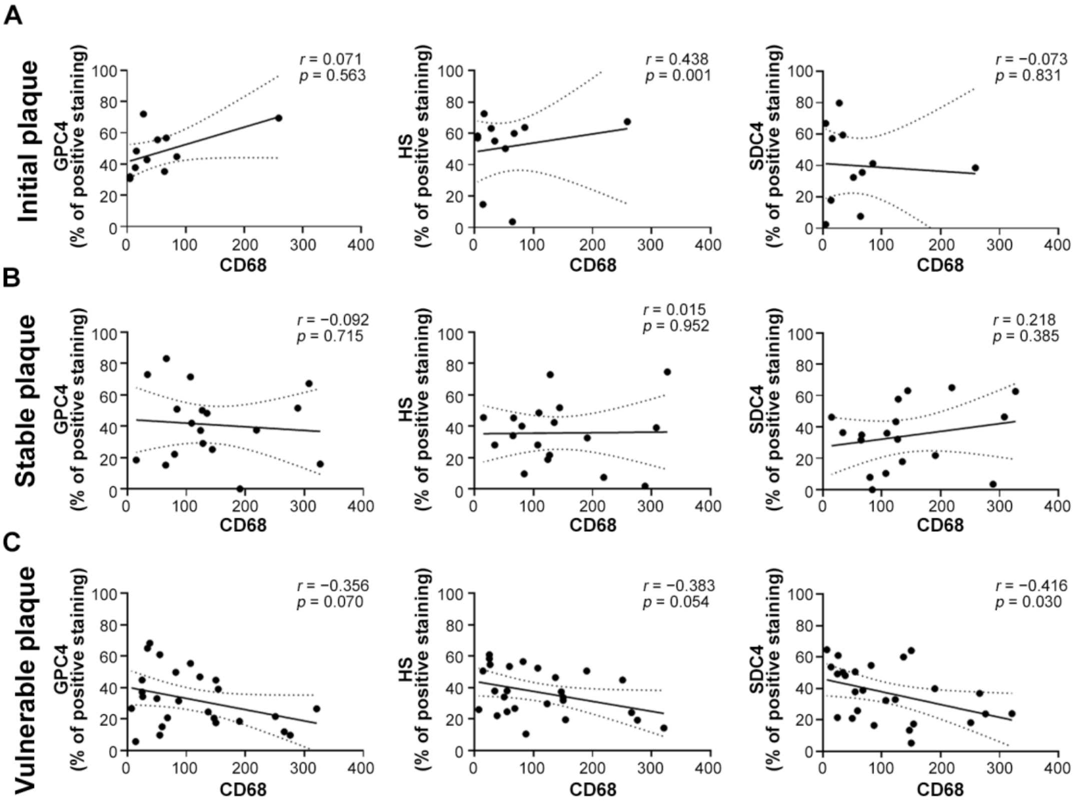

Figure S3. Correlation analysis between the GCX-components and CD68 positive cells in the more-inflamed PSs in initial (A), stable (B) and vulnerable (C) plaque sections

Pearson or Spearman correlation between GPC4, HS, and SDC-4 with CD68 were done in the more-inflamed PS region. Graphs showing simple linear regression with 95% confidence interval (dotted line). r: Pearson coefficient; ρ: Spearman coefficient, GPC4: glypican-4.

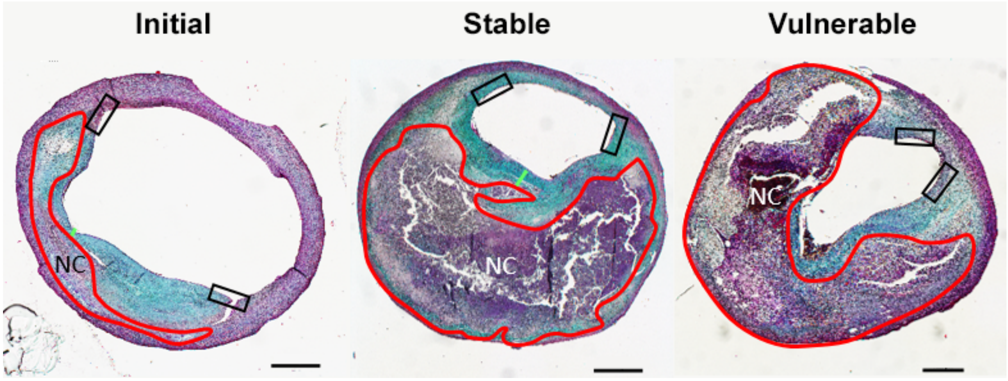

Figure S4. Representative pictures of plaque sections stained by modified Crossmon's Trichrom staining

Initial (left), stable (middle), and vulnerable (right) plaque sections. Images were taken at 20x magnification with an Olympus IX70 inverted microscope with NIS elements® software. The necrotic core (NC, red), the two plaque shoulders (black) and the thinnest part of the FC (green) are indicated. Scale bar = 1000 µm.
